# Motilitometer: A compact modular microscope for motility and photoresponse analysis of microorganisms

**DOI:** 10.1016/j.ohx.2026.e00788

**Published:** 2026-05-10

**Authors:** Alain Gervasi, Lindsey Schutz, Pierre Cardol, Patrick E. Meyer

**Affiliations:** a Genetics and Physiology of Microalgae, InBios/Phytosystems, University of Liège, 4 Chemin de la Valée, 4000 Liege, Belgium; b Bioinformatics and Systems Biology, InBios/Phytosystems, University of Liège, 4 Chemin de la Valée, 4000 Liege, Belgium; c BotaBotLab, InBios/Phytosystems, University of Liège, 4 Chemin de la Valée, 4000 Liege, Belgium

**Keywords:** Microscopy, Cell tracking, Motility, Phototaxis, 3D printing

## Abstract

In biology, cell motility is a valuable indicator, revealing how cells sense their environment, adapt to stimuli, and reflect their physiological state. In this paper, we present a modular microscopy system designed to study the motility of microorganisms. The setup is compact, assembled from low-cost 3D-printed and widely available components. It integrates programmable multispectral illumination to enable controlled photo stimulation and investigation of light-induced behavioral responses, including phototaxis. Initially developed for the investigation of photosynthetic microalgae, the system is easily adaptable to a wide range of motile microorganisms. It supports two complementary observation modes: a droplet-based chamber that minimizes sedimentation for cell speed analysis, and a deeper chamber that allows sedimentation, enabling quantitative assessment of motility fraction and orientation relative to directional light. The optical system provides a magnification of up to approximately 9×, sufficient for the precise centroid detection and tracking of single cells. This open-source, DIY (Do It Yourself) microscopy platform offers an accessible tool for photobiology, microbial ecology, and biophysics, helping democratize quantitative motility studies.

## Graphical abstract

**Figure:**
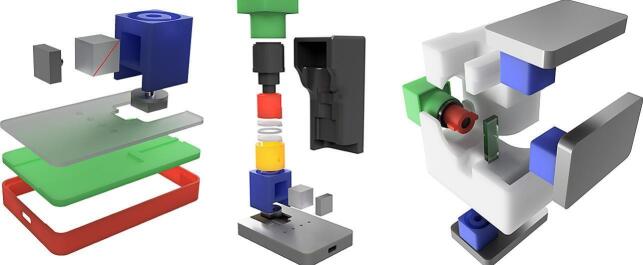

Specifications table.

**Table:** 

Hardware name	Motilitometer
Subject area	Biological sciences
Hardware type	Imaging tool
Closest commercial analog	No direct commercial equivalent combining programmable multi-wavelength illumination with quantitative motility tracking in free-swimming microorganisms
Open-source license	CERN-OHL-W v2
Cost of hardware	100-200€
Source file repository	https://zenodo.org/records/19616641

## 1 Hardware in context

Motility is a widespread strategy employed by unicellular microorganisms to explore their environment, optimize access to light and nutrients, escape unfavorable conditions or encounter potential symbiotic hosts [1], [2], [3], [4], [5], [6]. To achieve this, they have evolved diverse modes of locomotion, including flagella-driven swimming, ciliary beating, gliding and surface-associated motility, each adapted to specific ecological contexts [7]. The study of this behavior provides valuable insights into the physiological state of cells, including their energy balance, stress responses, and capacity to adapt to dynamic environments [8], [9], [10], [11], [12]. Beyond its ecological and physiological relevance, motility analysis is also increasingly used as a biosensing approach, where alterations in swimming behavior serve as a sensitive readout to detect heavy metals, pollutants, and other environmental stressors [13], [14], [15], [16].

The quantitative study of motility and light-dependent behaviors is a well-established field with dedicated systems for observing, measuring, and interpreting these processes having been developed over several decades [17], [18]. Over the years, a wide range of experimental strategies have emerged, reflecting both technological progress and evolving biological questions .

At the macroscopic scale, motility can be assessed by monitoring the accumulation or avoidance of cells along a controlled stimulus gradient (light or chemical). Such assays are straightforward to set up and analyze, and they can be easily parallelized across multiple conditions, making them well suited for rapid phenotyping [19], [20], [21], [22], [23], [24], [25]. Their readouts, however, are mostly qualitative, capturing population-level redistribution rather than individual trajectories or behaviors. Since typical swimming speeds are on the order of a few hundred micrometers per second, significant displacements generally require extended observation times. These methods are therefore not well adapted to detecting rapid, transient events such as photoshock, a rapid behavioral reaction triggered by abrupt increases in light intensity, during which the flagella reverse or stop their beating, causing a brief interruption or backward jump in swimming [26], [27], [28]. Nevertheless, this approach has been successfully used in various studies, contributing both to a better understanding of phototaxis and to the screening of mutants impaired in motility or photoperception [29], [30], [31], [32].

Microscopic observation overcomes these restrictions by enabling the direct tracking of individual cells, but it requires more sophisticated imaging setups and computational tools. Several open-source and low-cost microscopes have been developed recently [33], [34], [35], [36], [37], [38], [39], [40], [41], [42], [43], [44], [45], [46], [47], [48]. Although many were originally conceived for static imaging of samples mounted on slides rather than for motility analysis, they can nevertheless be adapted for this purpose. With appropriate modifications and experimental adaptations, they can be configured to observe freely moving cells under different conditions, either through modified microscope systems [45], [46], [47], [48], [49], [50], [51], [52], [53], [54] or custom optical bench setups [55], [56], [57], [58], [59], [60], [61], [62], [63]. In some cases, these developments have also been coupled with microfluidic platforms, enabling precise environmental control and the generation of well-defined chemical or light gradients while maintaining compatibility with high-resolution imaging [64], [65]. Other approaches have demonstrated the feasibility of compact, lens-free holographic devices [66], [67], [68], [69], [70], [71], [72], which are easy to build but provide only low-resolution structural information.

Such microscopic scale observations typically generate time-lapse from image sequences of motile organisms, where quantitative measurements are extracted by tracking cells across successive frames. A wide variety of tracking approaches have been developed, ranging from dedicated software packages to general-purpose particle-tracking algorithms [73], [74], [75], [76], [77]. While the underlying methods differ in implementation, the objective remains the same: to reconstruct trajectories and extract motility parameters such as trajectory shape, swimming speed, directionality, and turning behavior.

The system proposed in this paper, the Motilitometer, is an open-source platform specifically developed to implement digital video-microscopy and automated tracking within a light-controlled environment. By synchronizing image acquisition with a programmable illumination source, the device enables the quantification of light-dependent behaviors, ranging from steady-state swimming to rapid transitions like the photoshock response. To manage these assays, we developed a companion Python-based pipeline that handles hardware control and automates data analysis. This software extracts the motility parameters from population-scale to individual trajectory reconstruction within a single, transparent workflow.

## 2 Hardware description

The large variety of open-source microscopy systems [33], [34], [35], [36], [37], [38], [39], [40], [41], [42], [43], [44], [45], [46], [47], [48] is generally divided into two categories: Portable Field Microscopes (PFMs), optimized for mobility and basic diagnostics, and Multipurpose Automated Microscopes (MAMs), which offer research-grade flexibility but are often complex to replicate and maintain [48]. In this context, the proposed Motilitometer positions itself as a streamlined MAM, specifically engineered to limit reproducibility costs and complexity.

**Fig. 1:**
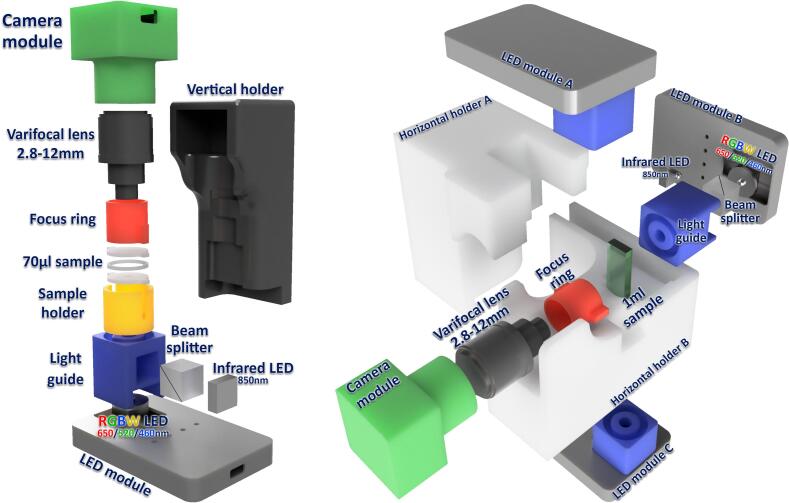
Modular design of the Motilitometer and supported observation geometries. [Left] Vertical configuration optimized for droplet-based measurements. The camera module equipped with a 2.8–12 mm varifocal lens and focus ring is mounted above the sample holder, which confines a ∼ 70 µL droplet between two slides. Illumination is provided from below by a combined RGBW (460, 520, 650 nm) and infrared (850 nm) LED module, whose outputs are merged by a beam splitter and guided to the sample via a light guide. [Right] Horizontal configuration designed for cuvette-based assays. A 1 mL sample contained in a 2 mm optical cuvette is positioned between the imaging module and the illumination modules. Directional stimulation and imaging are achieved using independent LED modules, while infrared illumination enables motility tracking without interference from visible stimulation light.

A common limitation of existing experimental setups is that they are frequently narrowly designed to address a specific biological question. This often results in fixed systems that are difficult to repurpose or require specialized infrastructures like microfluidics or advanced optical benches. In contrast, the Motilitometer is designed as an adaptive platform. Although optimized for motility and phototaxis, its modular architecture remains inherently versatile and is not restricted to a single task. Its optical and mechanical assemblies are designed for straightforward modification, enabling researchers to adapt it for routine monitoring, exploratory studies, or entirely new applications, all without requiring complex manufacturing instrumentation. This versatility is tangibly demonstrated by the system's native support for two complementary observation geometries (Fig. 1), allowing switching configurations based on the biological timeframe and physical constraints of the experiment:

● **Horizontal Configuration (Long-term & Directionality):** Cells are placed in a 2 mm quartz cuvette with the camera oriented perpendicularly. This setup integrates a dual-illumination system with independent sources positioned above and below the sample. This arrangement serves a dual purpose: it provides precise directional stimuli for orientation studies and enables sustained phototaxis monitoring. By periodically alternating the active light source, the system prevents phototactic organisms from accumulating at the cuvette boundaries, outside the field of view, thereby maintaining the population within the observation zone for extended periods.
● **Vertical Configuration (Fast Dynamics & Adhesion):** A culture droplet is confined between two PMMA slides separated by a 1 mm spacer. In this setup, non-motile cells rapidly leave the focal plane, effectively isolating the active swimmers. However, the focal plane can be adjusted to the bottom of the sample holder to image these sedimented cells, allowing for the quantification of the non-motile sub-population. The confined geometry creates a hydrodynamically stable environment with negligible fluid motion. This facilitates precise swimming speed measurements and allows for rapid sample preparation. This setup is ideal for capturing transient behaviors, such as photoshock responses or sudden trajectory changes. Since observations occur near the slide surface, it also enables the quantification of surface adhesion phenomena.

Both configurations preserve 3D free-swimming behavior, unlike traditional slide-coverslip preparations that restrict movement to a thin layer. By offering these interchangeable modes without requiring complex microfabrication, the hardware bridges the gap between simple diagnostics and high-end behavioral tracking.

The optical system is built around an inverted varifocal lens positioned directly in front of the camera sensor, achieving magnifications up to 9 × . Unlike fixed-focus lenses, the varifocal design allows magnification to be adjusted without exchanging objectives, further enhancing system versatility. While not as sophisticated as high-end fixed objectives, this configuration provides satisfactory performance consistent with previous studies [44] while maintaining both compactness and affordability.

Illumination is provided by a dedicated module that combines a high-power RGBW LED (650 nm, 520 nm, 460 nm and warm white), and an infrared imaging LED (830 nm), with their outputs merged through a beam splitter. The drivers support PWM-based current regulation ranging from 28 to 1500 mA with an input voltage of 2.9 to 6.1 V. This wide operating range allows the standard light sources to be easily substituted with a vast variety of alternative LED, enabling researchers to target specific wavelengths required by different biological models. Light intensity can be finely tuned (1–500µE m^−2^s^−1^), and the driver can be controlled via USB serial, Wi-Fi (MQTT), or a built-in web interface. Multiple drivers can be synchronized for complex stimulation protocols, offering high flexibility for experimental design.

Optical filters can be added in front of the camera to isolate a specific wavelength or to block the visible spectrum for exclusive infrared imaging. This feature allows motility to be monitored independently of the saturating visible light used for stimulation. The optical system was deliberately not optimized for quantitative fluorescence measurements, as the primary goal of the platform is motility tracking under controlled illumination rather than high-sensitivity fluorescence imaging. However, the camera sensor provides great quantum efficiency up to the near infrared region and a 10-bit image depth that allows for quantification of low-intensity light such as the weak autofluorescence of chlorophyll *a* which can help highlight or distinguish cells during observations.

This open hardware approach offers a strategic alternative to commercial research microscopes. Commercial systems typically operate as “black boxes,” characterized by undocumented proprietary hardware, expensive software licenses, and closed ecosystems that restrict the integration of custom peripherals or light sources. While commercial instruments undeniably offer superior optical resolution and mechanical robustness, their high cost prohibits the “fleet deployment” required for high-throughput screening. The Motilitometer accepts a calculated trade-off: it provides optical quality sufficient for quantitative behavioral analysis at a fraction of the price, prioritizing data sovereignty, accessibility, and open customization over absolute optical perfection.

Increasingly accessible entry-level commercial microscopes do not necessarily share the closed ecosystems or high costs of professional systems and could theoretically be easier to modify for custom experimental setups. However, they are still mainly optimized for standard flat-slide observations and lack the specialized sample holders required to maintain organisms in a 3D free-swimming environment. While optical filters can be added to change the light color, photobehavioral analysis requires multidirectional stimulation as well as precise, automated control over light intensity and temporal pulsing. Retrofitting the native electronics of a budget microscope to achieve this level of control can quickly become as complex as building a system from scratch, making a dedicated open-source platform a more straightforward approach.

To complement the hardware, the system integrates a Python-based acquisition and analysis pipeline designed with the same modular philosophy as the physical instrument. The software performs analysis at two levels: population-scale optical flow and individual single-cell tracking. Unlike proprietary commercial ecosystems that often obscure processing algorithms or require costly licenses for advanced analysis modules, this open-source stack grants full access to the source code. The tracking functions are encapsulated in independent classes, allowing researchers to inspect, modify, or integrate specific algorithms into their own workflows. This ensures that the system provides a complete, adaptable research tool characterized by full transparency regarding both instrumentation and data processing.

Our proposed hardware can help other researchers, by:

● Enabling quantitative observation of **swimming speed, trajectory geometry, phototaxis, adhesion, and photoshock responses** in motile microorganisms, with flexibility to adapt protocols to different species.
● Providing a low-cost platform to study **population-level dynamics** (e.g., motile vs. non-motile fractions, collective orientation under light) or **single-cell behaviors** (e.g., turning, reorientation).
● **Facilitating high-throughput screening:** The low cost and compact footprint allow for the deployment of “fleets” of parallel microscopes, enabling simultaneous data acquisition across multiple conditions.
● Providing an **educational tool** for teaching microscopy, light-organism interactions, and microbial ecology, thanks to its 3D-printed design and intuitive light control.
● Enabling improvements to our **customizable open framework**: researchers can add new light wavelengths, integrate microfluidics, or extend the Python analysis pipeline for novel experimental tasks.

All components are held by interchangeable 3D-printed holders, highlighting the modularity and adaptability of the system.

## 3 Design files summary

The files complete_assembly.f3d and.step (Table 1) contain all components properly arranged for use in either a vertical or horizontal configuration. All parts in STL format should be 3D printed, while those in DXF format are intended for laser cutting. The parts LED_driver_cap and Slide_separator are available in both formats and can be either printed or laser-cut. Components were 3D-printed in Prusament PETG using a Prusa i3 MK3S (0.4 mm nozzle, 0.2 mm layer height, 50% rectilinear infill). We also successfully printed the parts in PLA and observed no heat-induced deformation around the LED or driver modules. However, ABS is not recommended, as its shrinkage can negatively affect the tolerances required for the friction-fit design. Furthermore, the use of resin printers is not advised, as the resulting parts would lack the necessary flexibility for proper assembly.

**Table 1:** Design files summary.

**Design file name**	**File type**	**Open-source license**	**Location of the file**
Motilitometer_horizontal.step	STEP	CERN-OHL-W v2	https://zenodo.org/records/19616641
Motilitometer_horizontal.f3d	STL	CERN-OHL-W v2	https://zenodo.org/records/19616641
Motilitometer_vertical.step	STEP	CERN-OHL-W v2	https://zenodo.org/records/19616641
Motilitometer_vertical.f3d	STL	CERN-OHL-W v2	https://zenodo.org/records/19616641
Cam_box_A	STL	CERN-OHL-W v2	https://zenodo.org/records/19616641
Cam_box_B	STL	CERN-OHL-W v2	https://zenodo.org/records/19616641
Lens_holder	STL	CERN-OHL-W v2	https://zenodo.org/records/19616641
Focus_ring	STL	CERN-OHL-W v2	https://zenodo.org/records/19616641
Sample_holder	STL	CERN-OHL-W v2	https://zenodo.org/records/19616641
Light_guide	STL	CERN-OHL-W v2	https://zenodo.org/records/19616641
LED_driver_cap	STL	CERN-OHL-W v2	https://zenodo.org/records/19616641
LED_driver_side	STL	CERN-OHL-W v2	https://zenodo.org/records/19616641
LED_driver_base	STL	CERN-OHL-W v2	https://zenodo.org/records/19616641
Horizontal_cam_holder	STL	CERN-OHL-W v2	https://zenodo.org/records/19616641
Vertical_cam_holder	STL	CERN-OHL-W v2	https://zenodo.org/records/19616641
LED_driver_cap	DXF	CERN-OHL-W v2	https://zenodo.org/records/19616641
Slide	DXF	CERN-OHL-W v2	https://zenodo.org/records/19616641
Slide_separator	DXF	CERN-OHL-W v2	https://zenodo.org/records/19616641
LED_driver_firmware	INO	MIT	https://zenodo.org/records/19616641
Motilitometer_software	PY	MIT	https://zenodo.org/records/19616641

## 4 Bill of materials summary

Most components (Table 2) can be substituted with equivalent parts from other brands if those listed become unavailable from the referenced supplier. The microcontroller can also be replaced with unofficial clones of the original development board. The beam splitter does not need to be of high optical quality, as its purpose is simply to combine visible and infrared light. Since the LED intensity can be adjusted independently for each channel, the system can accommodate variable transmittance/reflectance. LED themselves can also be replaced with any model or wavelength suited to the project's specific needs. Finally, the camera used (a 10-bit monochrome global shutter model) can be replaced with a standard RGB webcam as long as the desired application does not require high framerates or low-light performance.

**Table 2:** Bill of materials.

**Designator**	**Component**	**Number**	**Cost per unit**	**Total cost**	**Source of materials**	**Material type**
3D printing filament	PETG filament	1	33€	33€	https://www.prusa3d.com/fr/product/prusament-petg-matte-black-1 kg/	Polymer
Microcontroller	Wemos D1	1	2.5€	2.5€	https://aliexpress.com/item/32631693796.html	Integrated circuit
LED driver	LD06AJSA/LD1500SB	4	1€	4€	https://aliexpress.com/item/1005005114675873.html	Integrated circuit
IR LED	850 nm 3 W diode	1	0.5€	0.5€	https://aliexpress.com/item/1005003037092401.html	Semi conductor
RGBW LED	RGBW 10 W LED	1	1€	1€	https://aliexpress.com/item/4000557258963.html	Semi conductor
Heatsink	14x14x6mm heatsink	2	0.1€	0.2€	https://aliexpress.com/item/32852107715.html	Aluminum
Beam splitter	10x10x10mm 5:5 beam splitter	1	5€	5€	https://aliexpress.com/item/1005008868545722.html	K9 glass
Varifocal lens	2.8–12 mm varifocal IR M12 lens	1	7€	7€	https://aliexpress.com/item/32467017612.html	Glass
IR longpass filter	RG830 LP 12.5 mm − 1 mm	1	35€	35€	https://www.edmundoptics.eu/p/schott-rg830-125mm-dia-1mm-thick-colored-glass-longpass-filter/44320/	Colored glass
Camera	See3Cam_20CUG	1	130€	130€	https://www.e-consystems.com/industrial-cameras/ov2311-monochrome-global-shutter-camera.asp	Integrated circuit
Alternative camera	OV9281	1	33€	33€	https://aliexpress.com/item/1005004586207588.html	Integrated circuit

## 5 Build instructions

### 5.1 LED driver

**Fig. 2:**
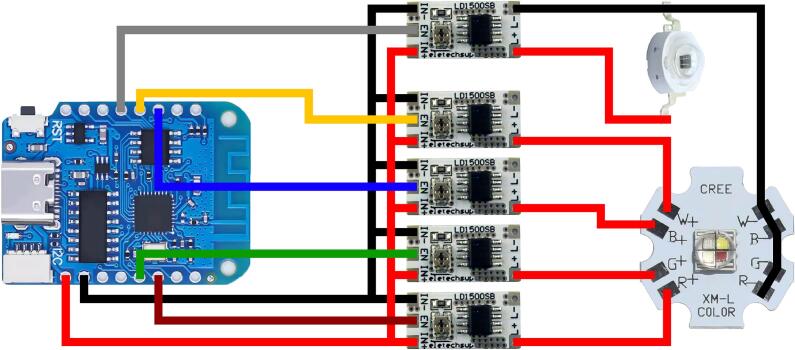
Electric diagram of the LED driver. [Left] The microcontroller (ESP8266) is connected to the current regulators [center] that independently drive the infrared LED [top right] and the RGBW LED [bottom right]. Red and black connections indicate the 5  V power supply bus connected to the ESP Vin and GND pins. Pulse-width modulation (PWM) control signals from the microcontroller's ESP8266 GPIOs are color-coded to match the corresponding LED channels: GPIO5 (red), GPIO4 (green), GPIO16 (blue), GPIO14 (white), and GPIO13 (infrared).

The LED illumination system is composed of an LED driver (Fig. 2) and a mechanical enclosure that maintains sample alignment with the microscope while shielding it from ambient light. It provides adjustable light intensity and wavelength, controlled via PWM by an ESP8266 microcontroller. The maximum intensity for each channel is first manually set using a potentiometer on the driver circuit. Subsequently, the PWM duty cycle is used to modulate the light between 0% and 100% of this predefined maximum. This dual-stage control allows light intensity to be adjusted between 1 and 500  µmol m^−2^s^−1^ with a resolution of approximately 2  µmol m^−2^s^−1^. The system supports remote control via USB (serial), Wi-Fi (MQTT), or a built-in graphical interface hosted by the microcontroller. The driver is USB-powered, requiring a 5 V/2A power supply. A module typically consumes 1 W when only the infrared light is turned on and reaches a maximum of 8 W when all 5 channels are operating at their maximum intensity.

To simplify assembly, a custom 5-channel integrated PCB design is also provided in the repository. While the standard assembly uses five individual, off-the-shelf drivers to ensure maximum accessibility, this alternative driver module is intended for users familiar with professional manufacturing services (e.g., JLCPCB or PCBWay). This integrated version allows for PCBA production, replacing the parallel wiring of separate drivers with a single, compact board. A dedicated 3D-printable housing (*Light_Driver_base_5Ch*) is provided for compatibility with this custom hardware.

**Fig. 3:**
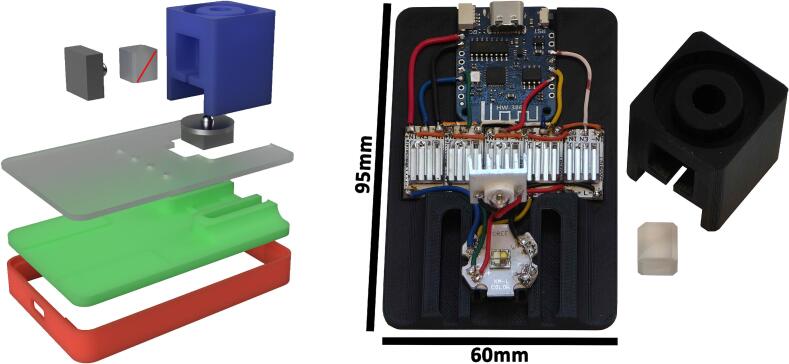
Assembly of the LED illumination system. [Left] Exploded view of the illumination module. Colored elements correspond to 3D-printed parts listed in the design file summary: [green] *LED_driver_base*, [red] *LED_driver_side*, [gray] *LED_driver_top*, and [blue] the light guide. The rectangular gray module represents the infrared LED, while the circular gray module corresponds to the RGBW LED. The red diagonal line indicates the reflective surface orientation of the beam splitter. [Right] Photograph of the assembled LED illumination module showing the electronic components mounted on the *LED_driver_base*.

The LED illumination system is assembled by first gluing LED current regulators on the *LED_driver_base*. The components operate well below their nominal ratings, so heating should be negligible. 10x10mm aluminum heatsinks can be added to the current regulator should high power output be required. The microcontroller can be soldered to the LED driver following the wiring diagram shown in Fig. 2. A 20 × 20 mm aluminum heatsink is attached beneath the RGBW LED using silicone thermally conductive adhesive glue (0.67 W/mK) or thermal adhesive pad, and the heatsink is then placed on the *LED_driver_base*. The LED can then be soldered to their respective LED drivers (Fig. 2). The light guide is then glued on top of the RGBW LED, and the beam splitter is inserted with the reflective surface facing the aperture, as indicated in Fig. 3. The infrared LED is then mounted on a heatsink in the same manner as the RGBW LED, soldered to its driver, and placed inside the light guide facing the beam splitter. Finally, the *LED_driver_side* and *top* panels can be installed. The maximum LED intensity can then be adjusted using the potentiometer on the current regulator modules.

### 5.2 Camera module

The camera board we used (See3CAM_20CUG) features a high-sensitivity global shutter sensor capable of capturing 10-bit monochrome images at up to 90 FPS. This configuration enables high-speed imaging under low-light conditions with minimal motion artifacts. An RGB camera can also be used, provided its sensor does not include an infrared cut filter, which is mandatory to produce accurate color reproduction. This filter is usually placed on the lens, but in some cases, it is integrated into the sensor module itself as a thin glass layer with a slight red tint. Since our system uses 850 nm IR illumination for imaging, the IR filter must be removed if present.

**Fig. 4:**
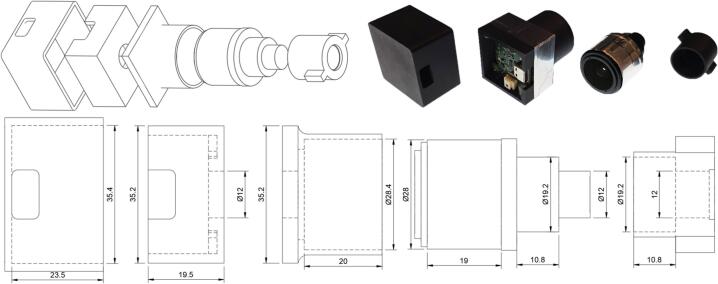
Camera assembly.

The camera module (Fig. 4) is assembled by gluing together two 3D-printed parts, *cam_box_B* and *lens_holder*. This two-part design facilitates 3D printing without support structures. Once assembled, the camera board is inserted into the housing and fixed using glue. The housing is then closed using the *cam_box_A* part. The varifocal lens is adjusted to achieve the desired magnification level depending on the size of the observed organisms, then secured using adhesive tape to prevent accidental focus shifts. The lens is then inserted into the *lens_holder* component. The 830 nm long pass filter is glued to the front of the *focus_ring*, which is then placed in front of the lens. The *focus_ring* holds in place by friction and can easily be swapped to change filters. As for the lens, a small amount of tape can be added to ensure stability.

It is worth noting that the total cost of the apparatus is primarily driven by the choice of camera. The structural and electronic components (excluding the imaging module) cost approximately 65€. While the complete reference setup costs around 200€, this could be reduced to under 100€ by using a standard webcam. Since imaging relies on infrared light, which does not affect the behavior of most photo-responsive organisms, high-intensity illumination can be used to compensate for the lower sensitivity of budget sensors.

We selected the See3CAM_20CUG (OV2311) for the reference design primarily as a conservative choice to maximize data control. It provides a global shutter with uncompressed RAW output and full manual control, avoiding unpredictable automatic adjustments and compression artifacts, alongside a 10-bit pixel depth for potential future light quantification. For users seeking a cost-effective compromise, the OV9281 module (detailed in Table 2) offers a middle ground, retaining a global shutter and a high frame rate (120 fps) at a significantly lower price point.

Although both models presented above feature global shutters to provide an extra margin of safety against motion artifacts, standard rolling-shutter webcams remain a fully viable alternative. Rolling shutter distortion remains marginal even under worst-case conditions. Given a small (10 µm diameter) and rapid cell (v=250μm/s) moving parallel to the scan axis at a low framerate (f=30FPS) within the constrained vertical field of view of the highest magnification level ($L_{\text{FOV}}=245\mu m$). The maximum relative velocity tracking error, calculated as the cell velocity divided by the product of the field of view and the frame rate, $E_{\text{rel}}=\frac{v}{L_{\text{FOV}}\cdot f}$ yields an error of approximately 3.4%. Similarly, the morphological distortion, defined as the distance travelled during the cell's specific readout window, $\Delta L=\frac{v\cdot L_{\text{cell}}}{L_{\text{FOV}}\cdot f}$ results in a maximum structural shift of only 0.34μm, falling well below the standard optical resolution of the device.

## 6 Operation instructions

### 6.1 LED driver

The LED driver can be controlled via three paths: a built-in web interface, MQTT over Wi-Fi or USB serial communication.

**Fig. 5:**
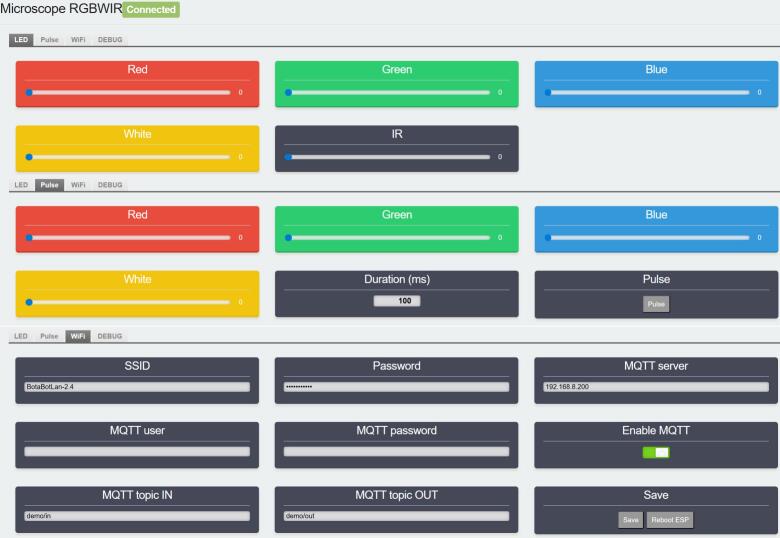
LED driver user interface.

The web UI is designed for quick and intuitive use. When powered on for the first time, the ESP automatically creates a Wi-Fi access point named “Microscope RGBWIR”. By connecting a smartphone or computer to this Wi-Fi network, a captive portal will open (or can be accessed via the IP address 192.168.4.1) featuring the control interface shown in Fig. 5, which is organized into three distinct sections. The first menu controls continuous illumination, allowing the intensity of each light channel to be adjusted individually. The second menu is dedicated to light pulses, enabling the user to trigger stimuli with precise intensity and duration. The third menu handles driver configuration, allowing users to set local Wi-Fi credentials and optionally define the address and password for an MQTT broker.

When operating in access point mode, the ESP acts as both a Wi-Fi router and a web server, and the interface is always accessible at the static IP address 192.168.4.1 if accessed from a device connected to the access point. However, when the driver is connected to a preexisting Wi-Fi network, the ESP behaves as a regular client and is assigned a dynamic IP address by the router. In that case, the web interface remains accessible, but users must connect to the specific IP assigned by the network. This address can usually be found via the router's administration panel, or it is automatically printed to the serial port at 115,200 baud when the ESP is powered on. Beyond simply displaying the IP address, this serial connection serves as a fallback interface. If Wi-Fi is unavailable, the device can be fully configured directly through the terminal.

While the web interface is convenient for quickly adjusting light settings, it does not allow scripting of complex light sequences or simultaneous control of multiple drivers. For these cases, the second control via MQTT over Wi-Fi can be used. MQTT (Message Queuing Telemetry Transport) [78] is a lightweight, publish/subscribe protocol widely used in IoT applications. It enables communication between devices through a central broker, allowing remote control, automation, and coordination of multiple LED drivers. By assigning specific subscription topics to each driver, it is possible to control them individually or simultaneously (e.g., by having multiple drivers subscribe to the same topic for synchronized operation, or to separate topics for parallel but independent control). The USB serial communication provides low-latency, synchronized control suitable for integration with custom software or real-time experiments. By default, the driver uses a baud rate of 115200.

The same command structure (Table 3) is used for both MQTT and USB serial control. The LED driver expects commands formatted as JSON objects, following the structure below:

**Table 3:** JSON command structure for LED driver control (via Serial or MQTT).

**Key**	**Type**	**Range**	**Description**
pulse	Integer	≥ 0 (ms)	Duration of light pulse
r	Integer	0–1000	Red channel intensity
g	Integer	0–1000	Green channel intensity
b	Integer	0–1000	Blue channel intensity
w	Integer	0–1000	White channel intensity
ir	Integer	0–1000	Infrared channel intensity

The “pulse” parameter triggers a temporary flash of the specified channels for the given duration in milliseconds, after which the previous light state is restored. If “pulse” is not included, the driver sets the light intensities as a constant output. All parameters are optional, only the desired channels need to be specified in each command.

Examples of commands:

● {“pulse”:100,“b”:1000}: emits a 100 ms pulse of blue light at full intensity.
● {“r”:100,“ir”:1000}: sets the red channel to 10% (actinic light) and infrared to 100% for imaging.

The driver configuration can also be edited via the serial interface. Available commands can be listed by typing help in the serial terminal, and the current configuration status can be viewed by typing info.

### 6.2 Command line interface

**Fig. 6:**
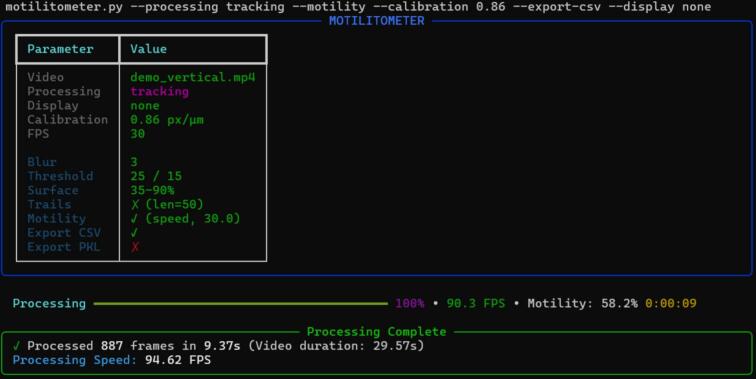
Example of command-line usage for motility analysis. Screenshot of the *motilitometer.py* interface during tracking-based motility processing. Algorithm parameters and calibration settings are defined at program startup via command-line arguments, and a summary of the used parameters is displayed before analysis. During processing, real-time information including progress status, instantaneous processing speed (FPS), current motility metrics, and elapsed time is continuously updated. A final summary reporting total processed frames and average processing speed is generated upon completion.

To complement the hardware, we developed a Python-based command line interface software designed for both real-time acquisition and retrospective analysis of pre-recorded videos (Fig. 6). The software features an intuitive interface enabling on-the-fly control of camera parameters, such as exposure and gain. Furthermore, it supports scripted measurement sequences, allowing for precise synchronization between LED illumination and video recording. The application incorporates the tracking methods described in section 7.4 to perform automated motility analysis. Built on a modular architecture, the core algorithms are encapsulated in independent classes for easy integration into external pipelines. All acquired footage are saved as standard*.mp4* videos. Measurement can be exported in the CSV format, while raw trajectory data is serialized to PKL files for subsequent analysis. While we are currently developing a dedicated graphical user interface (GUI) for subsequent publication, this universal video format ensures immediate compatibility with established 'no-code' environments. Users who prefer a visual interface can therefore seamlessly analyze their recordings using third-party software such as ImageJ/Fiji (via the TrackMate plugin), ToxTrac, Icy, among others.

### 6.3 Vertical setup (droplet measurement)

Sample preparation is performed by placing one of the slides (engraved side facing up) into the 3D-printed sample holder base. The laser-cut slide separator is then positioned on top. A 70  µL droplet of culture is carefully deposited at the center of the slide, within the engraved circle. The droplet is held in place primarily by surface tension. For optimal positioning, the slide must be completely clean and free of any contamination, otherwise the droplet could fail to form a stable hemispherical shape. The slide should be cleaned using water and soft paper and avoid using abrasive materials or solvents containing isopropanol, as those could damage the PMMA surface. Finally, a second slide (engraved side facing down) is placed on top to enclose the droplet, forming a thin and stable observation layer.

**Fig. 7:**
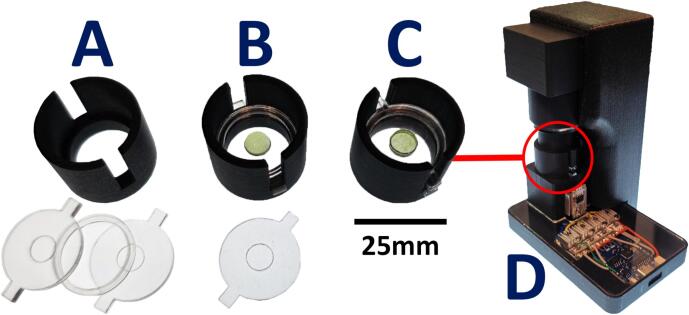
Sample preparation steps for the vertical imaging setup. [A] Components of the sample holder, consisting of a 3D-printed *sample_holder* base and two laser-cut components: a *slide_separator* and two PMMA slides (1  mm thickness). [B] Culture droplet placed on the bottom slide. [C] Assembled sample holder enclosing the droplet. [D] Assembled sample holder positioned on the illumination module, with the microscope placed above and secured using the *Vertical_cam_holder*.

The assembled sample container can then be placed onto the LED driver, in the dedicated recess of the 3D-printed holder, with the camera positioned above, as shown in Fig. 7. Focus adjustment is performed by rotating the sample holder clockwise or counterclockwise. This setup allows focusing through the full depth of the liquid column. It is therefore possible to focus on the top layer as easily as on the bottom one. The former is ideal for studying motility and cell adhesion because only motile cells can remain at the surface, while the latter allows a better study of sedimentation and accumulation of non-motile or settling cells.

### 6.4 Horizontal setup (cuvette measurement)

**Fig. 8:**
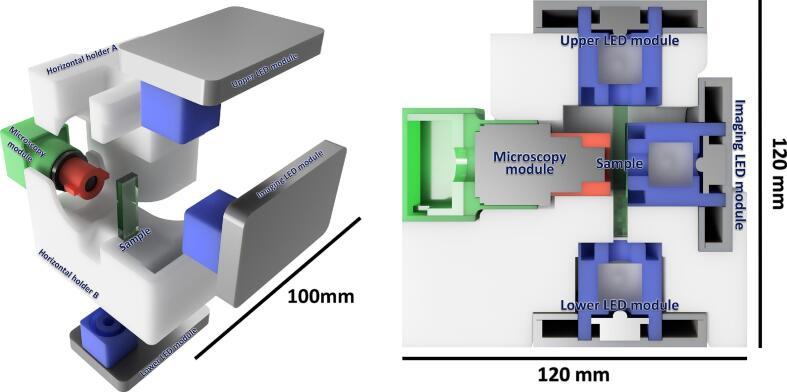
Assembly of the horizontal imaging setup. [Left] Exploded view of the horizontal configuration showing the relative positioning of the microscopy module, the sample holder, and the illumination modules. The sample is placed between the imaging LED module and the microscopy module, while additional upper and lower LED modules provide directional light stimulation. All components are mounted within two complementary 3D-printed supports (*Horizontal_holder A* and *Horizontal_holder B*). [Right] Top view of the fully assembled horizontal setup, illustrating the alignment of the microscopy module with the sample and the imaging LED module, as well as the orthogonal arrangement of the upper and lower LED modules.

The horizontal imaging setup shown in Fig. 8 is assembled by placing the “lower LED module”, “imaging LED module” and the “microscopy module” in their respective slots in the “horizontal holder B”. The sample holder, consisting of a 2 mm spectrophotometer cuvette, is filled with 1  mL of culture, leaving a small air bubble at the surface. Liquid injections using a micropipette may induce rotational fluid movement, which can disturb measurements. To neutralize these motions, the cuvette can be sealed at the top and briefly inverted, allowing the bubble to travel through the entire liquid column. This step helps homogenize the sample and dissipate residual fluid movement. Over time, convection currents may develop, or cells may fully sediment at the bottom of the cuvette. In such cases, the sample can be re-homogenized by repeating the inversion step. Once prepared, the cuvette is placed into the designated slot in “horizontal holder B” between the “microscopy module” and the “imaging LED module”. The secondary “upper LED module” is placed on the “horizontal holder A” which itself is placed on top of the sample and camera assembly. Focus is adjusted by moving the small horizontal protrusion of the focus ring. The focal plane allows observation of both faces of the cuvette. However, since this setup is primarily designed to observe freely swimming microalgae, it is preferable to focus near the center of the liquid column. After measurements, the cuvette can be emptied and rinsed.

## 7 Validation and characterization

### 7.1 Microscope module

**Fig. 9:**
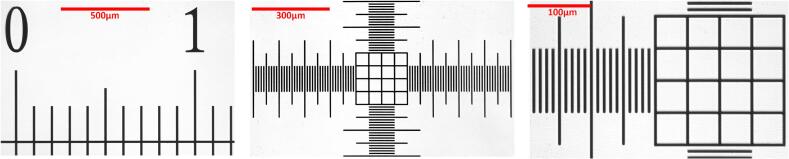
Calibration of the magnification level. Images of a calibration slide acquired at three magnification settings: [left] lowest magnification, [center], medium magnification and [right] maximum magnification. The calibration slide features 100 µm and 10 µm graduations, allowing determination of the pixel-to-micrometer conversion factor for each optical configuration.

The zoom level on Fig. 9 was adjusted by varying the focal length of the lens (from 2.8 to 12  mm) and by modifying the distance between the lens and the camera sensor (with a greater distance resulting in higher magnification). These measurements allowed the determination of image calibration factors in µm/pixel and confirmed the absence of optical aberrations. Notably, optical aberrations were minimized by recording at a resolution of 720p using a native 1080p sensor. This configuration creates a central crop, effectively excluding the lens periphery where distortions are typically most pronounced. Software-based correction remains an option if full sensor coverage is required.

These adjustments allowed for the determination of three distinct image calibration states:

• **High magnification (0.34 µm/px):** 8.8 × optical magnification with a FOV of 440 × 240 µm.
• **Medium magnification (0.86 µm/px):** 3.5 × optical magnification with a FOV of 1100 × 620 µm.
• **Low magnification (1.22 µm/px):** 2.5 × optical magnification with FOV of 1560 × 880 µm.

Medium magnification was selected for all subsequent experiments, as it provided the optimal balance between resolving detail and maintaining a sufficiently large FOV to track organisms ranging from 10 to 100 µm in size, which typically move at velocities of 50 to 250 µm/s.

Regarding the optical performance, while the nominal numerical aperture (NA) of an f/1.4 lens can theoretically reach 0.36, the effective NA in this inverted macro configuration, accounting for internal vignetting and aberrations at 850 nm, is estimated to be approximately 0.12. Based on the Rayleigh criterion ($r=\frac{0.61\lambda }{\text{NA}}$), the physical optical resolving power is limited to approximately 4.3 μm. Consequently, the system operates in an oversampled regime, which precludes the study of subcellular structures but remains fully sufficient for centroid-based motion analysis.

**Fig. 10:**
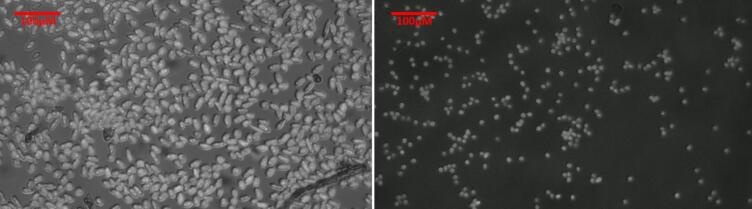
Chlorophyll *a* autofluorescence images of *Euglena gracilis* [left] and *Chlamydomonas reinhardtii* [right] acquired under using blue excitation light (460 nm) light using a 665 nm long pass emission filter.

In addition to bright-field imaging, which primarily relies on absorbance for contrast, the system can also be configured for fluorescence microscopy (Fig. 10). This is achieved by combining a specific excitation light source with an appropriate emission filter placed in front of the objective. As an example, live photosynthetic cells can be detected through the fluorescence emission of chlorophyll *a* in the 640–800 nm range upon excitation with blue light at 460 nm.

### 7.2 LED module

Using an external DSO18B20 (Dallas semiconductors) probe, the temperature of the LED chip, the LED driver, and the sample holder (in both horizontal and vertical configurations) were monitored to evaluate the system's thermal stability. The setup was subjected to continuous white light illumination at maximum intensity during 20 min, followed by a 10 min off-period to simulate an intensive, yet realistic operating scenario. This protocol extends beyond typical experimental exposures, which usually last a few minutes. The test was performed at an ambient room temperature of 24°C, relying entirely on passive cooling (no active fan assistance).

**Fig. 11:**
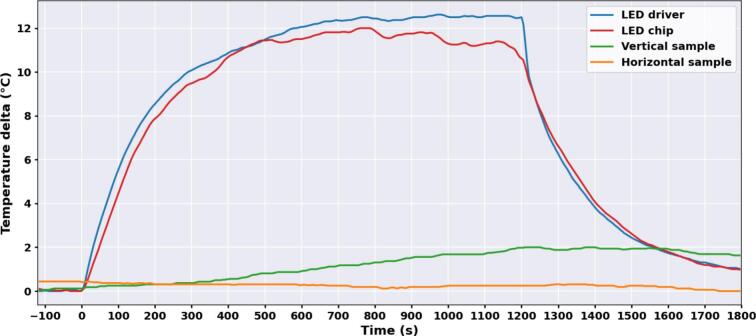
Thermal validation of the system during an intensive illumination cycle. Temperature variations (ΔT in °C) of the different system components over time during a 20 min illumination of white light at maximal intensity followed by a 10 min off period.

As illustrated in Fig. 11, the temperature of the electronic components stabilized with an increase of approximately 12°C (reaching ∼ 36°C). This is well below the maximum thermal limits of the components and is insufficient to induce any deformation of the plastic holder. Regarding the biological samples, a minor increase of ∼ 2°C was observed in the vertical setup (where light is concentrated on a small surface with a volume of only 70 µL), while the horizontal setup did not show any temperature increase. The slight warming observed in the vertical configuration remains acceptable and is unlikely to induce a major thermal shock. As this test demonstrates that temperature variations are minimal, continuous monitoring is not required for our experimental protocols and is therefore not included in the current design. Nevertheless, if other applications require it, users can easily add an external temperature probe using the available GPIO pins on the ESP microcontroller.

### 7.3 Culture protocol

Freshwater microalgae *Chlamydomonas reinhardtii CC-1010* strain and *Euglena gracilis* SAG1224-5/25 strain were obtained by first preparing pre-cultures in Tris-acetate-phosphate medium [79] under continuous light (30 µE m^−2^s^−1^) at 25°C, until reaching the exponential growth phase (approximately 2 days for *C. reinhardtii* and 7 days for *E. gracilis*). The use of an acetate-rich medium allowed for faster biomass accumulation. Total chlorophyll concentration was determined by methanol extraction following [80]. Cultures were then diluted in acetate-free minimal Tris-minimal-phosphate medium to a final chlorophyll concentration of 5  µg/mL. These autotrophic cultures were returned to continuous light (30 µEm^−2^s^−1^) for 24 h before being used for measurements.

Marine species*,* including *Tetraselmis striata* (RCC 130), *Tetraselmis convolutae* (isolated from *Symsagittifera roscoffensis*), *Tetraselmis* sp. (NIES-4478), and the protist *Rapaza viridis* (NIES-4477) were cultured in f/2-enriched seawater (G0154, Sigma-Aldrich) at a salinity of 34 g/L. Cultures of marine species were maintained at 25°C under a 12:12 h light/dark cycle. All assays were performed after 3 days of incubation.

### 7.4 Video analysis protocol

Swimming behavior was analyzed using custom Python scripts based on the OpenCV library. Two complementary approaches were used to analyze swimming behavior: a top-down method and a bottom-up method. The top-down approach is optimized for population-level analysis, particularly in videos with high cell density. It is robust to parameter variations, requires minimal tuning, and can be parallelized efficiently since it processes frame pairs independently. This makes it sensitive to short-term changes in swimming speed, but it does not provide continuous tracking of individual cells.

The bottom-up approach, on the other hand, enables continuous tracking of individual cells and is better suited for analyzing detailed trajectories and behaviors over time. It can capture orientation changes and path shapes, but requires more parameter adjustment, as detection depends on cell size, shape, and image brightness. Furthermore, continuous tracking prevents efficient parallelization, making it slower for high density cultures.

To ensure reliable trajectory reconstruction, cell concentration should allow for sufficient inter-individual spacing, preventing frequent physical contact or overlaps within the field of view. When cells are too close, the segmentation algorithm may fail to distinguish between individuals, merging them into a single detection, which leads to identity swaps or trajectory fragmentation. Users should therefore optimize the sample concentration to maintain a dilute regime where free-swimming behavior is preserved and individual silhouettes remain distinct.

#### 7.4.1 Top-down approach

The top-down analysis begins by computing the global motion of all pixels in the frame, which are subsequently grouped and filtered to extract the average displacement of individual cells. Dense optical flow [81] is computed between consecutive frames to generate matrices of motion angles and magnitudes. Low-magnitude vectors are removed by thresholding to reduce noise, while Canny edge detection [82] combined with area filtering is applied to isolate motile cells while excluding small debris and large aggregates. A motile cell typically spans several pixels in the image, where each pixel has its own optical flow vector. To characterize displacement, all vectors within the corresponding region are averaged to obtain a single mean motion angle and magnitude.

Drift caused by sedimentation or bulk fluid motion is estimated by vectors associated with non-motile cells. All displacement vectors are compared to identify those with similar direction and magnitude within a defined tolerance (typically 5-10°). The mean drift vector from this group is then calculated, and its inverse added to all other displacement vectors to correct for drift. In cases of strong positive or negative phototaxis, where most cells swim in the same direction, this method cannot be applied because collective motion would be misinterpreted as drift. Instead, drift is measured during a dark pre-exposure period, and the resulting correction vector is stored and applied throughout the experiment.

#### 7.4.2 Bottom-up approach

The bottom-up approach functions in reverse order compared to the previous one: it first isolates individual cells via segmentation and then links their positions across frames to build continuous trajectories. Adaptive thresholding [83] is applied to each video frame to produce a binary mask that isolates algae from the background. Contours are then detected using the Canny edge detection algorithm [82], and objects are filtered based on area. The upper and lower size thresholds are defined as percentages of the area distribution, typically excluding the 10% smallest and 10% largest objects to remove debris and cell aggregates. The centroid of each retained cell is then determined and tracked over time using the Norfair library [84], which assigns a unique ID to each detected point and links it across consecutive frames using a distance metric (typically Euclidean) combined with motion prediction. This ensures consistent tracking, even in the presence of brief occlusions or minor detection errors and allows continuous tracking of each cell throughout its appearance in the video.

After tracking, the resulting trajectories are analyzed to calculate swimming speed, swimming direction, and the proportion of motile versus non-motile cells, following the same criteria as in the first technique.

### 7.5 Motility assessment

Depending on the observation mode (vertical or horizontal), the appropriate motility metric differs, and the two analysis modes are complementary, reporting on distinct physiological properties. In both cases, swimming behavior is analyzed using the bottom-up tracking approach.

**Fig. 12:**
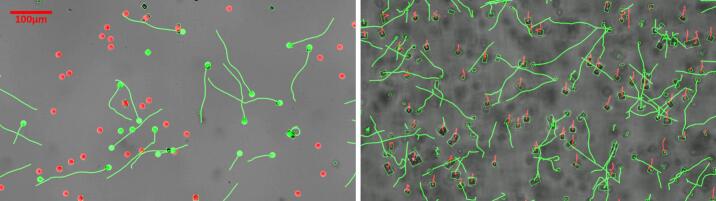
Motility analysis and classification of motile and non-motile *C. reinhardtii* cells. Representative examples of *Chlamydomonas reinhardtii* single-cell tracking and motility classification using two complementary criteria. [Left] Speed-based classification. Motile cells are identified based on their swimming speed and displayed in green, while non-motile or weakly moving cells are classified in red. [Right] Trajectory-based classification. Motile cells are identified based on trajectory geometry and directionality [green], whereas non-motile cells exhibit short, predominantly vertical trajectories associated with sedimentation [red]. Green lines represent reconstructed swimming trajectories.

In the **vertical configuration** (Fig. 12 left), the imaging plane is the top surface of the chamber. We observe swimmers and a subset of cells adhered to the wall. The tracking pipeline identifies all cells and measures individual speeds. Adherent cells have near-zero speed, enabling straightforward speed-based segmentation and a quick estimate of the motile fraction. Although this configuration does not provide an absolute motility fraction, it is well suited for comparative pre/post-treatment analyses and for probing cell-surface interactions.

In the **horizontal configuration** (Fig. 12 right), non-motile cells sediment through the field of view and may be misclassified as swimmers. However, they can be accurately segmented by their trajectories: non-motile organisms sediment at a uniform speed in a single downward direction while active swimmers exhibit a broad diversity of headings and velocities. Filtering by trajectory angle thus yields a reliable absolute motility percentage. This approach, however, is not suitable for adhesion studies, as measurements are taken in the water column rather than near the surface.

### 7.6 Optimal sample concentration

As discussed in Section 7.4, optimal tracking requires balancing cell density: excessive concentrations cause collision-induced artifacts, while low concentrations yield statistical variability due to under-sampling. To define optimal thresholds for two morphologically distinct organisms, exponential-phase *C. reinhardtii* and *E. gracilis* (in TAP medium) were quantified using a Beckman Coulter Z2 Counter. Serial dilutions from the undiluted cultures were then evaluated in both setups to identify the concentration range yielding the most consistent and reliable results across both observation modes.

**Fig. 13:**
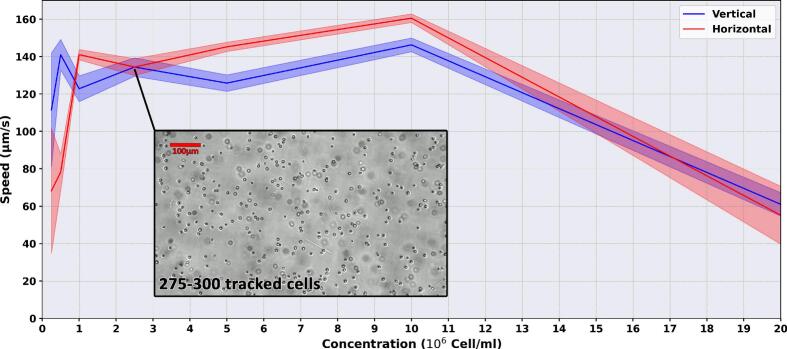
Effect of cell concentration on the measured motility of *C. reinhardtii*. Measured swimming speed (µm/s) across varying cell concentration for both vertical (blue line) and horizontal (red line) configurations. Shaded areas represent the standard deviation derived from 6 technical replicates (30-second video recordings) per concentration point.

As illustrated in Fig. 13, the Motilitometer is most effective within a concentration range of 10⁶ to 5 × 10⁶ cells/ml for tracking *C. reinhardtii*, providing an ideal balance between statistical robustness and tracking accuracy. At densities below 10⁶cells/ml, the scarcity of cells produces high statistical variability, rendering the measurements unreliable. Conversely, as the concentration increases beyond 5 × 10⁶cells/ml, cell-to-cell collisions become frequent. This crowding induces identity-swapping artifacts within the tracking algorithm, artificially inflating the perceived swimming speed. Beyond 10^7^ cells/ml, the extreme proximity and constant physical collisions between organisms severely hinder their movement, leading to a sharp decline in their actual swimming speed.

**Fig. 14:**
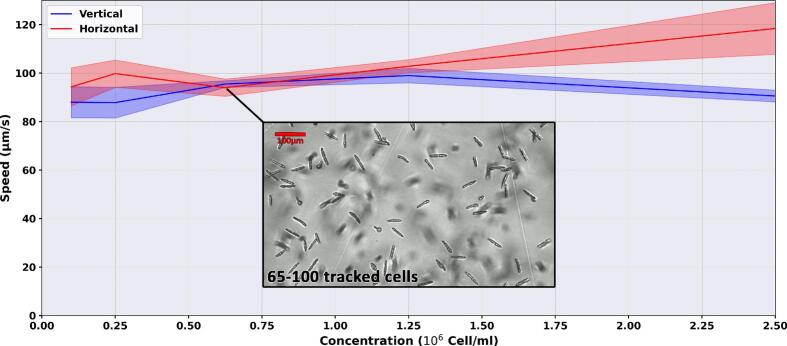
Effect of cell concentration on the measured motility of *E. gracilis*. Measured swimming speed (µm/s) across varying cell concentration for both vertical [blue line] and horizontal [red line] optical configurations. Shaded areas represent the standard deviation derived from 6 technical replicates (30-second video recordings) per concentration point.

For *E. gracilis*, Fig. 14 shows that the system operates reliably within a concentration range of 10^5^ to 10⁶ cells/ml. Within this window, speed measurements in the vertical setup remain remarkably stable. This is likely due to the larger size (∼100 µm) and lower maximum density of these organisms, combined with their rectilinear swimming pattern, which minimizes tracking variability and collisions. However, beyond 1.25 × 10⁶cells/ml, the horizontal setup exhibits an increase in measured speed. Due to the large cross-sectional area of *E. gracilis*, high densities of cells generate significant visual noise as they overlap, which the algorithm may misinterpret as rapid movement. This effect is particularly pronounced when the continuous sedimentation of cells through the field of view clutters the background. In contrast, the vertical setup effectively filters this noise: non-motile or sedimenting cells fall to the bottom of the chamber and exit the focal plane, maintaining a cleaner signal for the active population.

### 7.7 Photoperception and photoshock assessment

To illustrate the vertical setup and LED driver control, we performed photoshock response assays on *C. reinhardtii*. This configuration is ideal for such experiments as it minimizes fluid disturbances and allows precise observation of rapid behavioral changes, in this case brief interruptions in swimming. These conditions are also well suited to validate the precision of both the timing and intensity control of the LED illumination system. Swimming motility was quantified in real time between consecutive frames using the top-down optical flow approach, providing a direct measure of instantaneous speed dynamics during the assays.

**Fig. 15:**
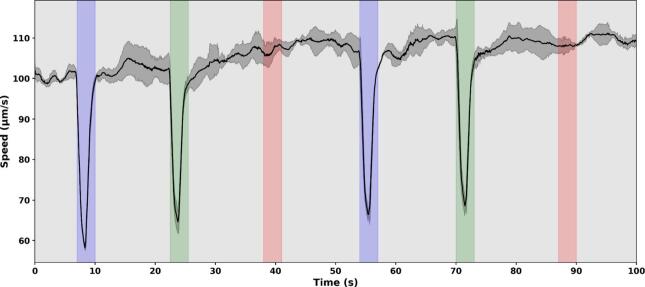
Photoshock response of *C. reinhardtii* to brief saturating light pulses. Cells were exposed to 100 ms light pulses at an intensity of 500 µEm^-2^s^−1^ delivered at 10 s intervals. Pulses were applied sequentially at 460 nm [blue], 520 nm [green], and 650 nm [red], as indicated by the colored shaded regions. The Y-axis shows the average swimming speed of the cells, quantified using optical flow analysis of image sequences recorded at 30 FPS. Shaded areas around the mean trace indicate variability across frames.

*C. reinhardtii* samples were exposed to 100  ms light pulses of varying wavelengths at an intensity of 500 µE m^-2^s^−1^, and swimming speed was monitored throughout the experiment (Fig. 15). A clear photoshock response was observed under blue and green light, whereas red light elicited no detectable change in swimming behavior. This is fully consistent with the known spectral sensitivity of Chlamydomonas channelrhodopsins, which are inactive beyond 600  nm [85]. These results confirm both the specificity of the behavioral response and the system’s capacity to deliver precise, wavelength-selective light stimulation.

**Fig. 16:**
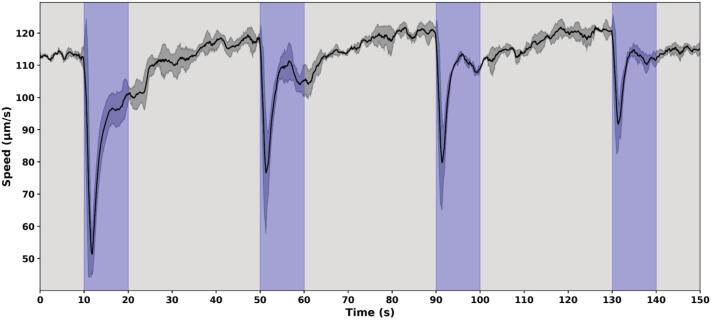
Response of *Chlamydomonas reinhardtii* to long, saturating blue light pulses. Cells were exposed to 10 s blue light (460 nm) at an intensity of 500 µE m^−2^ s^−1^ delivered every 40 s. Illumination periods are indicated by blue shaded regions. The Y-axis shows the average swimming speed of the cells, quantified using optical flow analysis of image sequences recorded at 30 FPS.

In the setup shown in Fig. 16, cells were exposed to a prolonged blue light illumination lasting 10 s to examine whether the duration of the photoshock response increases with longer stimuli or remains inherently short. We also tested whether repeated illuminations lead to a progressive reduction in response. In all cases, the response duration remained relatively stable at around 5 s, independently of the illumination duration.

Similar durations were observed in Fig. 15 experiment. Swimming activity resumed even while the light was still on, indicating that photoshock is a transient response triggered by the onset of intense illumination, rather than maintained by its continued presence. This behavior appears to be a short-term, fixed response that briefly halts movement when cells are suddenly exposed to strong light, likely preventing them from entering the illuminated area. It may act as an initial protective mechanism, distinct from longer-term behavioral strategies such as reorientation or escape (negative phototaxis). The gradual decline in response intensity after repeated flashes supports this interpretation, as prolonged swimming inhibition would be counterproductive under continuous light exposure.

### 7.8 Phototaxis assessment

The horizontal setup is well suited for measuring the proportion of motile cells and analyzing their orientation or swimming direction within the water column. The experiments presented in the following section will demonstrate this type of observation and showcase the simultaneous control of multiple LED drivers, with one dedicated to imaging and another used to apply directional light stimulation. Motility analysis is performed using the top-down optical flow approach, with non-motile cells filtered out based on their speed.

**Fig. 17:**
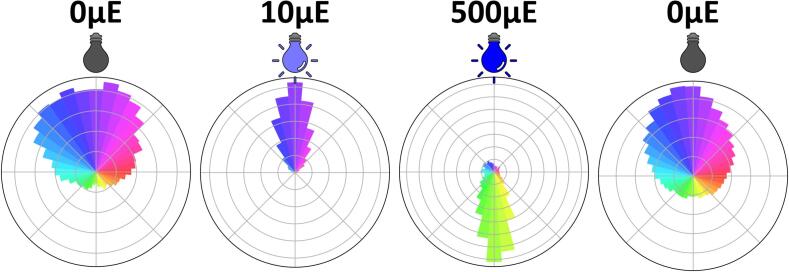
Analysis of positive and negative phototaxis in *Chlamydomonas reinhardtii*. Polar plots showing the swimming orientation of *C. reinhardtii* cells under different light conditions. From left to right: dark-acclimated cells (T0), exposure to low-intensity blue light (10 µEm^−2^s^−1^, T = 30 s), exposure to high-intensity blue light (500 µEm^−2^s^−1^, T = 60 s), and recovery of light removal (dark, T = 90 s). The light source is positioned at the top of each plot. Bar length represents the relative frequency of swimming directions.

In darkness, *Chlamydomonas reinhardtii* cells exhibit upward-biased swimming, although their trajectories remain relatively scattered. A small proportion of cells still swim downward. This upward bias may be explained by a gravity-induced vertical orientation. Upon exposure to low-intensity blue light at the surface of the sample, nearly all cells reorient toward the light source. When exposed to high-intensity light during 30 s, a rapid reversal of swimming direction is observed (Fig. 17). Once the light is turned off, the swimming behavior gradually reverts to the pattern observed initially under dark acclimation.

**Fig. 18:**
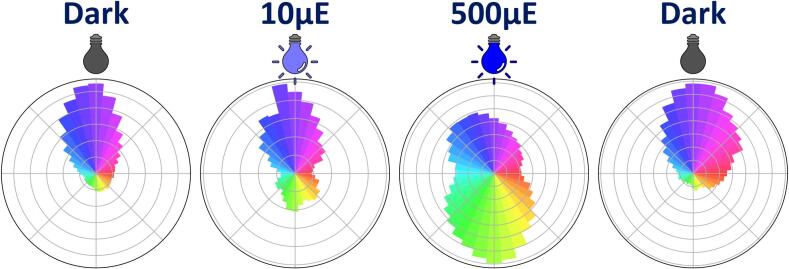
Analysis of positive and negative phototaxis in *Euglena gracilis*. Polar plots showing the swimming orientation of *E. gracilis* cells under different light conditions. From left to right: dark-acclimated cells (T0), exposure to low-intensity blue light (10 µEm^−2^s^−1^; T = 30 s), exposure to high-intensity blue light (500 µEm^−2^s^−1^, T = 30 s), and recovery after light removal (dark, T = 60 s). The light source is positioned at the top of each plot. Bar length represents the relative frequency of swimming directions.

Unlike *C. reinhardtii*, which uses two flagella for directed steering in response to light, *E. gracilis* possesses two flagella, but only one is used for propulsion. As a result, its orientation relies on a less precise tumbling mechanism [86]. In darkness, *E. gracilis* cells naturally orient upward (Fig. 18), when exposed to intense light, cells exhibit an erratic swimming behavior, characterized by frequent directional changes and a pronounced tendency to sediment. Unlike *C. reinhardtii*, no coordinated reorientation or directed escape away from the light is observed. However, the disorganized downward movement may reflect a form of negative phototaxis as a disruption of the gravitaxis. Once the light stress is removed, cells resume upward swimming.

Altogether, these results highlight species-specific phototactic strategies and illustrate how the same experimental setup can reveal distinct behavioral regimes.

### 7.9 Measurement of the swimming speed of various species

To assess the versatility of the Motilitometer, we quantified the swimming speeds of multiple aquatic microswimmers under dark conditions (Table 4). Measurements were performed using the vertical observation configuration combined with the top-down analysis pipeline. The selected species span a broad range of cell sizes, morphologies, and swimming behaviors. The system reliably captured distinct motility regimes across these organisms, demonstrating that the platform can be applied to diverse aquatic microswimmers beyond a single reference model.

**Table 4:** Swimming speed of selected microorganisms under dark conditions. Average swimming speed (µm/s) and corresponding standard deviation (SD) measured in dark-acclimated cultures using the Motilitometer system.

Strain	Average speed	SD
*Chlamydomonas reinhardtii* CC1010	94 µm/s	4.6
*Chlamydomonas reinhardtii* phot1	92 µm/s	14.2
*Chlamydomonas reinhardtii* CC3663	75 µm/s	1
*Chlamydomonas reinhardtii npq4*	46 µm/s	2.8
*Tetraselmis convolutae*	246 µm/s	11
*Tetraselmis Striata*	151 µm/s	10
*Tetraselmis NIES 4478*	65 µm/s	1.1
*Rapaza Viridis*	164 µm/s	1.2
*Euglena gracilis*	86 µm/s	3.1

### 7.10 Conclusion

We built and tested a compact, low-cost, open-source microscopy setup that reliably measures microbial motility. Across multiple scenarios, it demonstrated automated, programmable illumination (multi-wavelength/intensity) and flexible control, enabling experiments that probe speed, directionality, phototaxis, adhesion, and related physiological parameters. The two complementary observation modes enable producing video files that can be analyzed with standard computer-vision pipelines (optical flow or single-cell tracking). Our results show that the instrument works as intended, is easy to assemble from off-the-shelf parts, and is straightforward to operate. These attributes make the platform an accessible tool for routine monitoring, automated screening or hypothesis-driven studies in photobiology and microbial ecology. Beyond photobiology, the platform can be readily adapted to study motility responses to chemical, mechanical, or thermal stimuli. Together, the open hardware designs, firmware, and analysis scripts released with this work lower both the technical and financial barriers to quantitative motility assays, and should greatly ease adoption, replication, and further development of the system by the community.

## CRediT authorship contribution statement

**Alain Gervasi:** Writing – original draft, Visualization, Validation, Software, Methodology, Investigation, Formal analysis, Data curation, Conceptualization. **Lindsey Schutz:** Validation. **Pierre Cardol:** Writing – review & editing, Supervision, Resources, Project administration. **Patrick E. Meyer:** Writing – review & editing, Supervision, Funding acquisition.

## Declaration of competing interest

The authors declare that they have no known competing financial interests or personal relationships that could have appeared to influence the work reported in this paper.
